# Non-monotonic thickness dependence of MoS_2_–Sb contact resistance driven by bandgap and interlayer transport

**DOI:** 10.1371/journal.pone.0360960

**Published:** 2026-10-16

**Authors:** Luhao Liu, Zhengyi He, Yixiang Li, Shengsheng Lin, Songlin Li

**Affiliations:** National Laboratory of Solid-State Microstructures, School of Electronic Science and Engineering, Nanjing University, Nanjing, China; Universidad Autónoma de Querétaro: Universidad Autonoma de Queretaro, MEXICO

## Abstract

Contact resistance at metal–two-dimensional semiconductor interfaces is a major factor limiting the performance of emerging two-dimensional electronic devices. Understanding how material thickness influences contact behavior is therefore essential for device optimization. Here, we systematically investigate the layer-dependent contact resistance of MoS_2_–Sb interfaces using MoS_2_ field-effect transistors with different layer numbers. Contact resistance was extracted using the transmission line method and compared for devices fabricated on SiO_2_ and HfO_2_ gate dielectrics. The results reveal a pronounced non-monotonic dependence of contact resistance on the MoS_2_ layer number. As the layer number increases from one to three, the contact resistance decreases and reaches a minimum value of 308 Ω·μm for trilayer MoS_2_, while further increasing the thickness leads to a rise in resistance. Analysis of the electronic band structure and interlayer transport mechanisms indicates that this behavior originates from the competition between bandgap reduction and increasing interlayer transport resistance. In the few-layer regime, bandgap narrowing lowers the Schottky barrier and reduces contact resistance, whereas in thicker layers the growing interlayer resistance dominates carrier transport. These findings provide important insight into thickness-dependent contact physics in two-dimensional semiconductors and offer practical guidance for optimizing contact engineering and channel thickness in next-generation electronic devices.

## 1 Introduction

The continuous evolution of semiconductor technology follows the predicted trajectory of Moore’s Law, driving continuous breakthroughs in the miniaturization of integrated circuit dimensions and performance improvement [1]. However, as the process nodes enter the nanoscale and even sub-nanoscale, traditional silicon-based materials and device structures are increasingly facing severe challenges from physical limits [2]. Short-channel effects, increased leakage current caused by quantum tunneling, a sharp rise in power density, and heat dissipation problems have become fundamental bottlenecks restricting the further development of silicon technology [3,4]. Against this background, the discovery and rise of two-dimensional (2D) materials have opened up a new dimension for the development of semiconductor technology in the post-Moore era [5,6].

Since the emergence of graphene with its unique Dirac cone energy band structure and extremely high carrier mobility, a series of 2D layered materials with rich electrical, optical, and mechanical properties have successively come into the research spotlight [7,8]. Due to their atomic-scale thickness, absence of surface dangling bonds, excellent mechanical flexibility, and stack-ability, these materials show great prospects for device miniaturization and multi-functional integrated applications. Among them, 2D semiconductor materials with appropriate bandgaps, especially transition metal dichalcogenides (TMDCs), are regarded as ideal channel materials for constructing next-generation low-power, high-performance logic transistors [9,10].

Among numerous TMDCs, transition metal sulfides led by MoS_2_ stand out in both fundamental research and device applications [11]. They possess excellent electrical properties such as high mobility and on/off ratio, as well as unique physical characteristics like indirect-to- direct bandgap transition and atomic-level phase transition [12,13]. Therefore, efforts have been continuously made to use MoS_2_ as the channel material for various electronic devices. However, compared with silicon-based devices that have undergone decades of process optimization, the contact resistance of two-dimensional semiconductor devices is often several orders of magnitude higher due to the existence of Fermi-level pinning [14]. This severely limits the drive current and device transconductance, becoming the main bottleneck restricting their performance.

To reduce the contact resistance of two-dimensional semiconductors, researchers have attempted various strategies. First, doping MoS_2_ to reduce the Schottky barrier width or enable tunneling by increasing the carrier concentration [15,16]. Second, designing novel contact geometries, such as edge contacts or in – plane heterojunction contacts, to increase the contact area or change the injection path [17]. Third, using van der Waals integration technology to transfer pre-fabricated electrodes onto the MoS_2_ surface, avoiding interface damage and impurity introduction caused by traditional deposition processes [18,19]. Although these methods have achieved certain progress, they often have limitations. For example, doping may introduce scattering centers or damage the material stability [20]; complex contact structures increase the process difficulty and uncontrollability; and the reliability and large-scale fabrication of van der Waals contacts still face challenges [21]. Therefore, a deep understanding and fundamental regulation of the intrinsic interface physics between MoS_2_ and metals, especially revealing the evolution law of contact resistance with material parameters [22] (such as the number of layers) [23] are crucial for realizing high-performance and reproducible two-dimensional devices.

In response to the above challenges, this study aims to uncover the rules of the contact resistance between semi-metallic antimony (Sb) electrodes and MoS_2_ with different layers through precise device fabrication, systematic electrical characterization, and in-depth theoretical analysis, and clarify the dominant physical mechanisms behind it. The choice of semi-metallic Sb as the contact material is based on its unique electronic structure: Sb has a zero-bandgap characteristic and an extremely low density of states near the Fermi level [24], which can effectively suppress the formation of metal-induced gap states, thereby significantly weakening the Fermi level pinning effect and creating conditions for obtaining a low and tunable Schottky barrier [25,26] Compared with other low melting point semi-metals such as bismuth (Bi) and tin (Sn), Sb has a higher melting point (631°C), showing better thermal stability and compatibility with traditional semiconductor processes, making it more practical [26].

Although significant progress has been achieved in reducing the contact resistance of MoS_2_ transistors through contact metal engineering, interface doping, edge contacts, and van der Waals integration, systematic experimental studies on the thickness dependence of semi-metallic Sb contacts remain scarce. Most previous investigations have concentrated either on optimizing contact materials or improving interface quality, while relatively little attention has been devoted to understanding how the evolution of the MoS_2_ electronic structure with layer number influences carrier injection at Sb contacts.

In this work, we systematically investigate the contact resistance of Sb-contacted MoS_2_ field-effect transistors with thicknesses ranging from monolayer to five layers using the transmission line method (TLM). Devices were fabricated on both conventional 90 nm SiO_2_ and high-κ 10 nm HfO_2_ gate dielectrics to evaluate the robustness of the observed thickness dependence under different electrostatic environments. The experimental results consistently reveal a pronounced non-monotonic dependence of contact resistance on MoS_2_ thickness, with the minimum contact resistance obtained for trilayer MoS_2_.

To further elucidate the underlying physics, the experimental observations are interpreted by considering the competition between the reduction of the Schottky barrier arising from thickness-dependent band structure evolution and the increasing interlayer transport resistance in multilayer MoS_2_. The results provide practical guidance for optimizing both channel thickness and contact engineering in next-generation two-dimensional electronic devices employing semi-metallic Sb contacts.

Table 1 summarizes representative contact resistance values reported for MoS_2_ and related two-dimensional semiconductor devices using different contact materials and engineering strategies. As shown, contact resistance varies considerably depending on the contact configuration, interface engineering approach, and device architecture. Although several studies have demonstrated low-resistance contacts, systematic investigations of the thickness-dependent contact behavior of semi-metallic Sb electrodes remain limited. In this work, we not only achieve a low contact resistance of 308 Ω·μm for trilayer MoS_2_ but also demonstrate that the contact resistance exhibits a pronounced non-monotonic dependence on MoS_2_ thickness. By correlating the experimental results with the evolution of the electronic band structure and interlayer transport characteristics, this work provides additional physical insight into the contact formation mechanism of MoS_2_-Sb interfaces beyond simply reporting a low contact resistance.

**Table 1:** Comparison of reported contact resistance values for MoS_2_ and related 2D semiconductor contact technologies.

Contact strategy/ material	Semiconductor	Minimum contact resistance (Ω·μm)	Extraction method	Reference
2D/2D ohmic contact	MoS_2_	~300	TLM	[15]
TiO_2_ interfacial n-doping	MoS_2_	~2400	TLM	[16]
Indium alloy van der Waals contact	WS_2_	Near-ideal band offset (contact resistance not explicitly reported)	TLM	[19]
Indium alloy van der Waals contact	WSe_2_	Near-ideal band offset (contact resistance not explicitly reported)	TLM	[19]
Thickness-dependent metal contact	MoS_2_	68,000	TLM	[23]
Fe-doped contact	Monolayer MoS_2_	489	TLM	[10]
Undoped monolayer contact	Monolayer MoS_2_	~117,000	TLM	[10]
Ni contact	Few-layer MoS_2_	~500	TLM	[13]
**Semi-metal Sb contact (This work)**	**Trilayer MoS**_2_	**308**	**TLM**	**This work**

Note: Reported contact resistance values were extracted from the respective references under different device architectures, fabrication processes, carrier concentrations, and measurement conditions. Therefore, the comparison is intended to provide a representative benchmark rather than a direct one-to-one performance comparison.

The remainder of this paper is organized as follows. Section 2 describes the experimental methodology, including the preparation of MoS_2_ flakes, device fabrication processes, structural characterization, and electrical measurement techniques used to extract the contact resistance. Section 3 presents the experimental results and discusses the thickness-dependent behavior of the MoS_2_–Sb contact resistance based on transmission line measurements under different gate dielectric conditions. The physical mechanisms responsible for the observed non-monotonic trend are analyzed in terms of band structure evolution and interlayer transport resistance. Finally, the main findings and their implications for contact engineering in two-dimensional semiconductor devices are summarized in the conclusion.

## 2 Methodology

### 2.1 Preparation of MoS_2_ flakes

MoS_2_ flakes with different thicknesses were mechanically exfoliated from bulk crystals using adhesive tape and transferred onto highly doped silicon substrates with a 90 nm SiO_2_ layer. Optical microscopy was used to locate suitable flakes with uniform geometry. The MoS_2_ layer number was initially identified by optical contrast under an optical microscope and subsequently verified by atomic force microscopy (AFM). Representative AFM images together with the corresponding height profiles were used to confirm the layer-number assignment from monolayer to five-layer MoS_2_ prior to device fabrication. Samples with layer numbers ranging from one to five layers were selected for device fabrication.

### 2.2 Fabrication of bottom-gated devices on 90 nm SiO_2_

To investigate the contact resistance between Sb electrodes and MoS_2_ channels, transmission line method (TLM) structures were fabricated on the selected MoS_2_ flakes.

First, the MoS_2_ channels were patterned using electron beam lithography (EBL). After defining the channel regions, reactive ion etching (RIE) was performed to remove excess material and form channels with uniform width. Subsequently, a second EBL process was used to define the electrode patterns of the TLM structures with multiple channel lengths.

After lithography, Sb/Ag metal contacts were deposited by electron-beam evaporation. Sb contacts were deposited by thermal evaporation under a base pressure of **2 × 10^−5^ Pa**. To improve film quality, the first **10 nm** of Sb was deposited at a rate of **0.15 Å/s**, followed by deposition of the remaining **30 nm** at **0.35 Å/s**. Subsequently, a **40 nm Ag** capping layer was deposited at **0.35 Å/s** to prevent oxidation and reduce the series resistance of the contact. A 40 nm Sb layer was first deposited to form the electrical contact with MoS_2_, followed by a 40 nm Ag layer serving as a protective capping layer to prevent oxidation of Sb. The lift-off process was then carried out in acetone to remove the photoresist and complete the electrode fabrication. Fig 1 shows a three-dimensional schematic of the single-layer MoS_2_ device.

**Fig 1:**
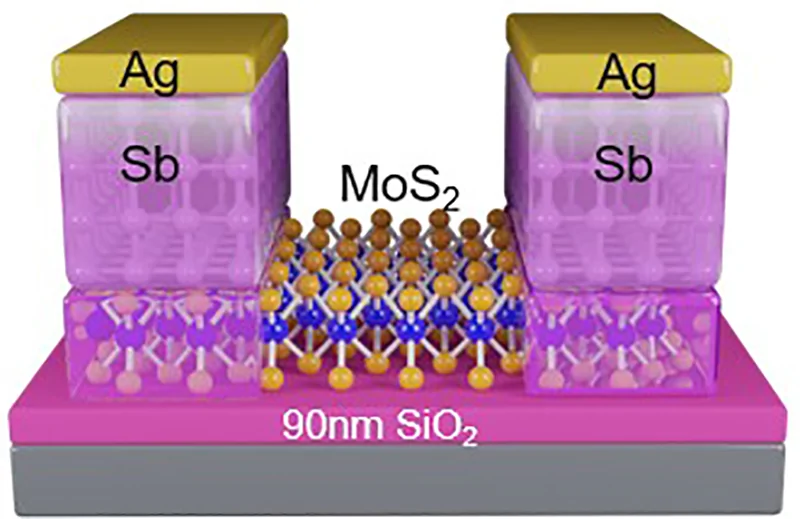
Three-dimensional schematic of the single-layer MoS_2_ device.

### 2.3 Fabrication of bottom-gate devices with 10 nm HfO_2_ dielectric

To enhance the gate electrostatic control and increase carrier concentration in the MoS_2_ channel, devices incorporating a high-κ gate dielectric were also fabricated.

The high-κ HfO_2_ gate dielectric was deposited by atomic layer deposition (ALD) using H_2_O as the oxidant at a deposition temperature of 150 °C for 80 cycles. Prior to electron-beam lithography, the samples were annealed at 120 °C, followed by annealing in an H_2_/Ar atmosphere for 60 min to remove residual contaminants and improve device quality. First, Ni/Pd (5/10 nm) metal layers were deposited onto a silicon substrate by electron-beam evaporation to serve as the bottom gate electrode. Subsequently, a 10 nm HfO_2_ dielectric layer was deposited on top of the gate electrode using atomic layer deposition (ALD).

MoS_2_ flakes were then transferred onto the prepared HfO_2_ substrate using a poly (propylene carbonate) (PPC) assisted transfer method. After the transfer process, the fabrication of electrodes and TLM structures followed the same procedures used for the SiO_2_-based devices, including EBL patterning, RIE channel definition, and Sb/Ag electrode deposition. Fig 2 shows a three-dimensional schematic of the monolayer MoS_2_ field-effect transistor with 10 nm HfO_2_ as the bottom gate dielectric.

**Fig 2:**
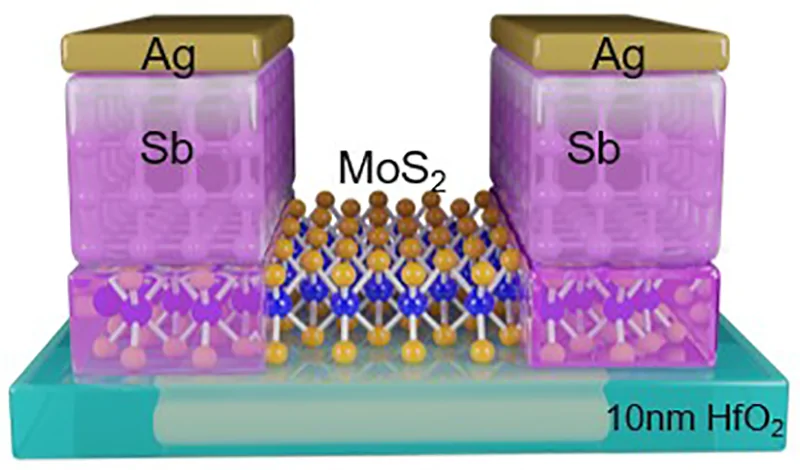
Schematic of the monolayer MoS_2_ field-effect transistor with 10 nm HfO_2_ as the bottom gate dielectric.

The morphology and thickness of the mechanically exfoliated MoS_2_ flakes were characterized by AFM. Representative AFM images and corresponding height profiles were acquired to verify the layer-number assignment from monolayer to five-layer MoS_2_ prior to device fabrication. The AFM measurements confirmed well-defined terraces and progressively increasing step heights with increasing layer number, providing reliable structural verification of the exfoliated flakes. The MoS_2_ layer number was initially identified by optical contrast and subsequently confirmed by AFM height-profile measurements.

### 2.4 Structural characterization

The morphology and geometry of the fabricated devices were characterized using optical microscopy and scanning electron microscopy (SEM). SEM imaging was used to accurately determine the electrode spacing and calibrate the actual channel lengths of the TLM structures. This calibration is necessary because slight deviations between the designed and actual channel dimensions can occur during the electron beam lithography and development processes.

### 2.5 Electrical measurements

Electrical characterization of the fabricated devices was carried out using a probe station (CRX-6.5K, Lake Shore) under high vacuum conditions with a pressure below 10^−6^ Torr. All measurements were performed at room temperature.

A Keithley 2636B digital source meter was used to apply voltages and record electrical signals. The transfer characteristics (*I*_ds_–*V*_g_) were measured at a fixed drain voltage of *V*_ds_ = 0.1 V for devices with different channel lengths.

For devices fabricated on the HfO_2_ dielectric, hard probes were used during the measurement to ensure reliable electrical contact with the metal bottom gate through the dielectric layer. All devices were measured immediately after fabrication to minimize the influence of environmental contamination or interface degradation.

### 2.6 Contact resistance extraction using the transmission line method

The contact resistance between Sb electrodes and MoS_2_ was extracted using the transmission line method (TLM) [27]. In this method, multiple electrodes with different channel lengths were fabricated on the same MoS_2_ flake.

The total resistance of the device can be expressed as:


$$\begin{array}{c} R_{total}=\text{2}R_{C}+R_{SH}\frac{L}{W} \end{array}$$ (1)

where $R_{C}$ represents the contact resistance, $R_{SH}$ is the sheet resistance of the MoS_2_ channel, L is the channel length, and W is the channel width.

By plotting the total resistance $R_{total}$ as a function of channel length L, a linear relationship is obtained. The intercept of the fitted line with the resistance axis corresponds to twice the contact resistance (2*R*_c_), while the slope provides the sheet resistance $R_{SH}$ of the channel. Representative examples of the TLM extraction procedure are already shown in Fig 3. For each device, the measured total resistance was plotted as a function of the calibrated channel length, and linear least-squares fitting was performed to extract the contact resistance and sheet resistance. The fitting quality was evaluated using the linear fitting equation, Pearson’s correlation coefficient (*r*), coefficient of determination (*R²*), adjusted *R²*, residual statistics, and the standard errors of the fitted parameters.

**Fig 3:**
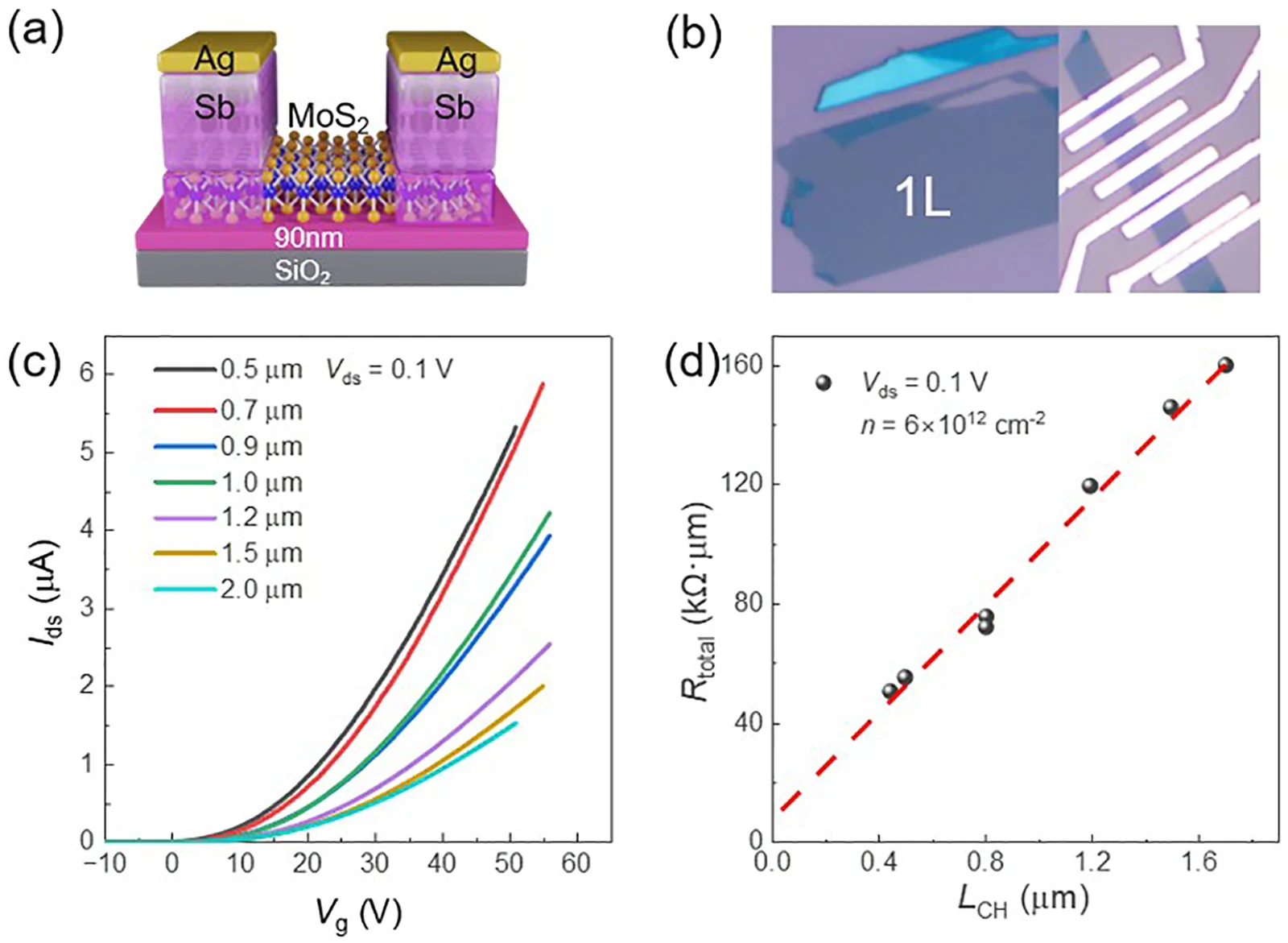
Influence of the number of MoS_2_ layers with 90 nm SiO_2_ on transfer-characteristics and contact resistance. (a) Schematic diagram of TLM geometry. (b) Optical images of the sample before and after device fabrication. (c) Transfer characteristics (I_ds_-V_g_) measured at V_ds_ = 0.1 V for channels of different lengths. (d) Example of parameter extraction based on the transmission line method at a carrier concentration of n = 6 × 10^12^ cm^-2^.The intercept of the fitted line with the R_total_ axis corresponds to 2R_C_. The sheet resistance R_SH_ is extracted from the slope of the line.

The fabricated transmission line method (TLM) structures consisted of multiple channel lengths between identical contact pads. The actual channel lengths used in the contact resistance extraction were determined from the fabricated device geometry.

## 3 Results and discussion

To systematically investigate the influence of the number of MoS_2_ layers on contact resistance, we first prepared MoS_2_ samples with different numbers of layers on 90 nm SiO_2_ and constructed multi-terminal field-effect transistors for the TLM test. The semi-metallic antimony (Sb) was selected as the electrode material, and an Ag protective layer was in-situ evaporated on the Sb layer to suppress its surface oxidation. Fig 3(a) shows a three-dimensional schematic of the single-layer MoS_2_ device, and Fig 3(b) provides optical microscopy images of the device before and after fabrication. All the fabricated devices were placed on a probe station for electrical testing, and the transfer characteristic curves at different channel lengths were measured, as shown in Fig 3(c).

During the testing process, we found that there was a certain difference between the actual channel length and the designed value due to the deviation in the EBL and development processes. Therefore, a SEM was used to accurately calibrate the channel size. Based on the transmission line method and the normalized contact resistance extraction formula [28]:


$$\begin{array}{c} R_{total}=W\cdot \frac{V}{I_{ds}} \end{array}$$ (2)

$$\begin{array}{c} R_{total}=\text{2}R_{C}+R_{CH}=\text{2}R_{C}+R_{SH}L_{CH} \end{array}$$ (3)

In the formula, *W* is the channel width, *R*_C_ is the normalized contact resistance, *R*_CH_ is the channel resistance, *R*_SH_ is the sheet resistance, and *L*_CH_ is the channel length. According to the above model method, as shown in Fig 3(d), the contact resistance *R*_C_ of a single-layer MoS_2_ device with 90 nm SiO_2_ as the back gate is 3387 Ω·μm at a carrier concentration of 6 × 10^12^ cm^−2^. This value is significantly higher than the typical value of the MoS_2_-Sb contact resistance reported in the literature [25,26].

Compared with the 10 nm HfO_2_ devices, the 90 nm SiO_2_ devices exhibit higher contact resistance because the thicker dielectric produces weaker electrostatic gate coupling and therefore a lower induced carrier concentration under the same gate bias. Consequently, the absolute contact resistance values differ between the two dielectric platforms. Nevertheless, both SiO_2_ and HfO_2_ devices consistently exhibit the same non-monotonic dependence of contact resistance on MoS_2_ thickness, indicating that the proposed transport mechanism is robust over different electrostatic operating conditions.

To further verify the applicability of the transmission line method, linear relationships between the total resistance and channel length were obtained for all devices, with coefficients of determination *R²* ranging from 0.989 to 0.999 as shown in Table 2. These results confirm that the TLM assumptions remain valid over the investigated channel-length range and that the extracted contact resistance values are reliable.

**Table 2:** Summary of the linear least-squares fitting parameters obtained from the transmission line method (TLM) analysis for Sb-contacted MoS_2_ field-effect transistors fabricated on 90 nm SiO_2_ and 10 nm HfO_2_ gate dielectrics.

Substrate	Layer	No. of TLM channel lengths	Intercept (Ω)	Slope (Ω μm^−1^)	Pearson’s *r*	*R²*	Adj. *R²*
**90 nm SiO**_2_	1L	7	6774.59 ± 460.10	90918.58 ± 4233.32	0.99462	0.98928	0.98713
	2L	4	5700.15 ± 1883.06	25206.43 ± 1620.36	0.99589	0.99180	0.98770
	3L	5	5064.86 ± 1170.53	32548.26 ± 1205.24	0.99834	0.99669	0.99559
	4L	5	4706.31 ± 492.92	35740.44 ± 1915.83	0.99992	0.99983	0.99979
	5L	5	4020.27 ± 610.83	36962.68 ± 2438.64	0.99977	0.99955	0.99940
**10 nm HfO**_2_	1L	7	1470.14 ± 386.33	12826.86 ± 187.80	0.99946	0.99893	0.99872
	2L	5	1300.75 ± 644.72	17089.20 ± 978.51	0.99674	0.99349	0.99023
	3L	5	1282.46 ± 269.73	10461.12 ± 253.66	0.99586	0.99175	0.99009
	4L	5	616.65 ± 295.31	14544.79 ± 258.34	0.99764	0.99529	0.99451
	5L	5	1434.56 ± 334.91	10828.93 ± 170.76	0.99907	0.99814	0.99768

Although both carrier mobility and sheet resistance depend on the MoS_2_ thickness, TLM enables these effects to be separated from the contact resistance. In the linear TLM model, the intercept corresponds to the total contact resistance, whereas the slope represents the sheet resistance of the MoS_2_ channel. Consequently, thickness-dependent variations in carrier mobility primarily influence the channel resistance and therefore appear in the fitted sheet resistance rather than in the extracted contact resistance. The excellent linearity obtained for all TLM fittings (*R²* = 0.989–0.999) confirms that the contact resistance extraction is reliable and that the contact and channel contributions are effectively decoupled.

To enhance the gate control ability and carrier concentration, we further employed atomic layer deposition technology to fabricate 10 nm HfO_2_ as the high-κ gate dielectric on the device surface. Meanwhile, to reduce the influence of organic residues on the contact interface during the transfer process, poly (propylene carbonate) (PPC) was selected to replace polydimethylsiloxane (PDMS) as the transfer support layer. Fig 4 (a) presents a three-dimensional schematic of the monolayer MoS_2_ field-effect transistor with 10 nm HfO_2_ as the bottom gate dielectric. The optical images of the sample before preparation and after transfer and completion of the preparation are shown in Fig 4 (b). The electrical test results (Fig 4 (c)) indicate that when the carrier concentration is increased to 2.5 × 10^13^ cm^-2^, the contact resistance drops to 735 Ω·μm (Fig 4 (d)). This is in good agreement with the results reported in the literature, verifying the reliability of the experiment and the extraction method [25,26].

**Fig 4:**
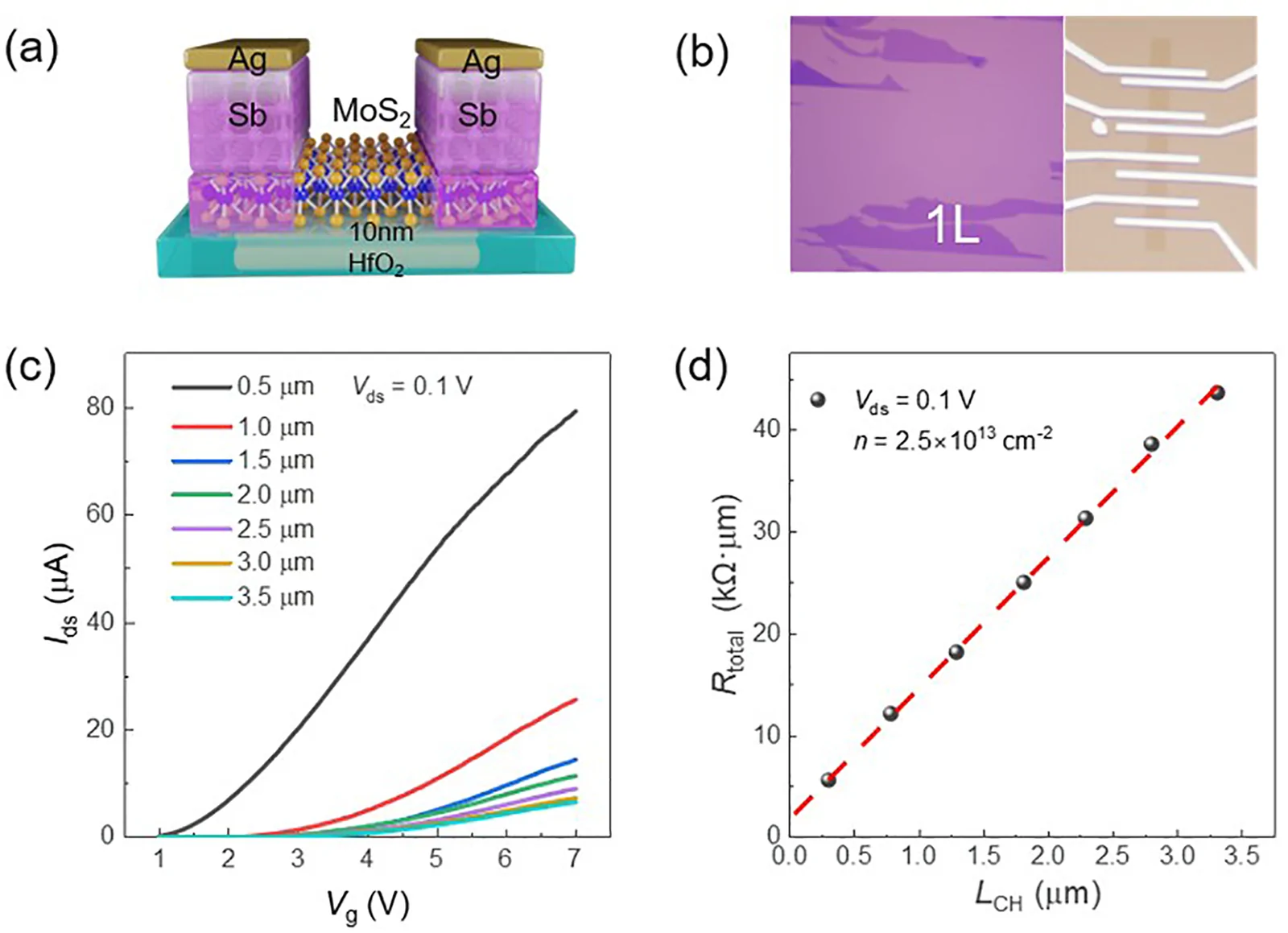
Influence of the number of MoS_2_ layers with 10 nm HfO_2_ on transfer-characteristics and contact resistance. (a) Schematic diagram of the TLM geometry. (b) Optical images of the sample before and after device fabrication. (c) Transfer characteristics (I_ds_-V_g_) measured at V_ds_ = 0.1 V for channels of different lengths. (d) Example of parameter extraction based on the TLM at a carrier concentration of n = 2.5 × 10^13^ cm^-2^.The intercept of the fitted line with the R_total_ axis corresponds to 2R_C_. The sheet resistance R_SH_ is extracted from the slope of the line.

Fig 5 presents representative optical/AFM images and the corresponding AFM height profiles of mechanically exfoliated MoS_2_ flakes with thicknesses ranging from monolayer to five layers. The optical images reveal uniform, well-defined flakes suitable for device fabrication, while the AFM height profiles exhibit progressively increasing step heights consistent with increasing layer number. These measurements verify the layer-number assignment used throughout this work and confirm the high structural quality of the exfoliated MoS_2_ prior to electrical characterization. Although AFM characterization verifies the layer thickness and morphology of the exfoliated MoS_2_ flakes, complementary techniques such as cross-sectional TEM, Raman spectroscopy, and XPS could provide further insight into the atomic-scale interface structure and chemical interactions at the MoS_2_-Sb interface. These investigations will be pursued in future work.

**Fig 5:**
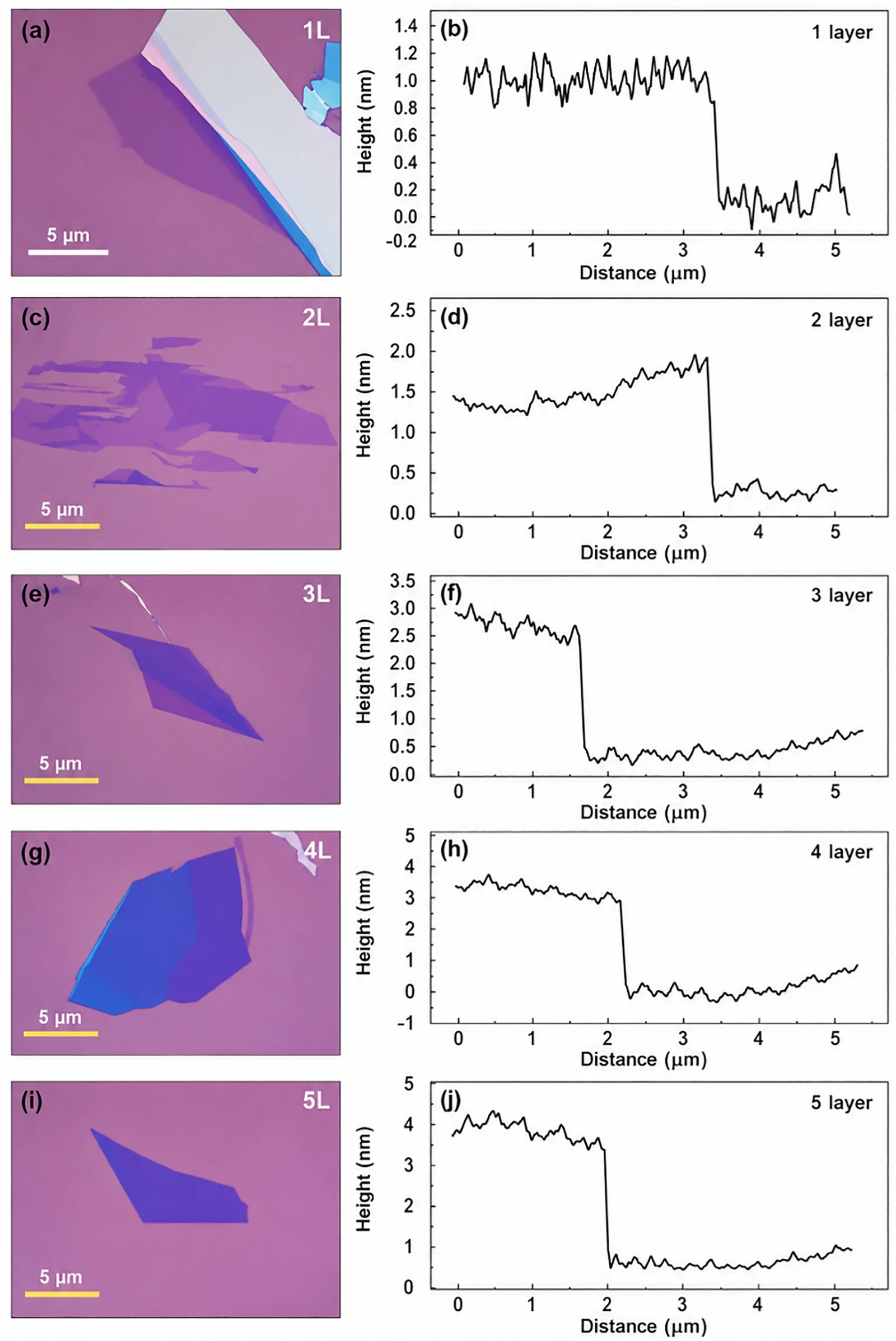
AFM characterization of mechanically exfoliated MoS_2_ flakes.

To reveal the evolution law of contact resistance with the number of layers, we systematically fabricated and tested 2- to 5- layer MoS_2_ devices (see Figs 6 and 7 for optical and electrical results, respectively). As shown in Fig 8 (a), in the SiO_2_ back – gated devices, when the number of layers increases from 1 to 3, the contact resistance decreases from 3387 Ω·μm to 2010 Ω·μm; when the number of layers further increases to 5, the resistance rises back to 2532 Ω·μm. A similar trend is also observed in the HfO_2_ gate dielectric devices (Fig 8 (b)). The 3-layer MoS_2_ device exhibits the lowest contact resistance (308 Ω·μm), and the contact resistances of the 1-layer and 5-layer devices are 735 Ω·μm and 717 Ω·μm, respectively.

**Fig 6:**
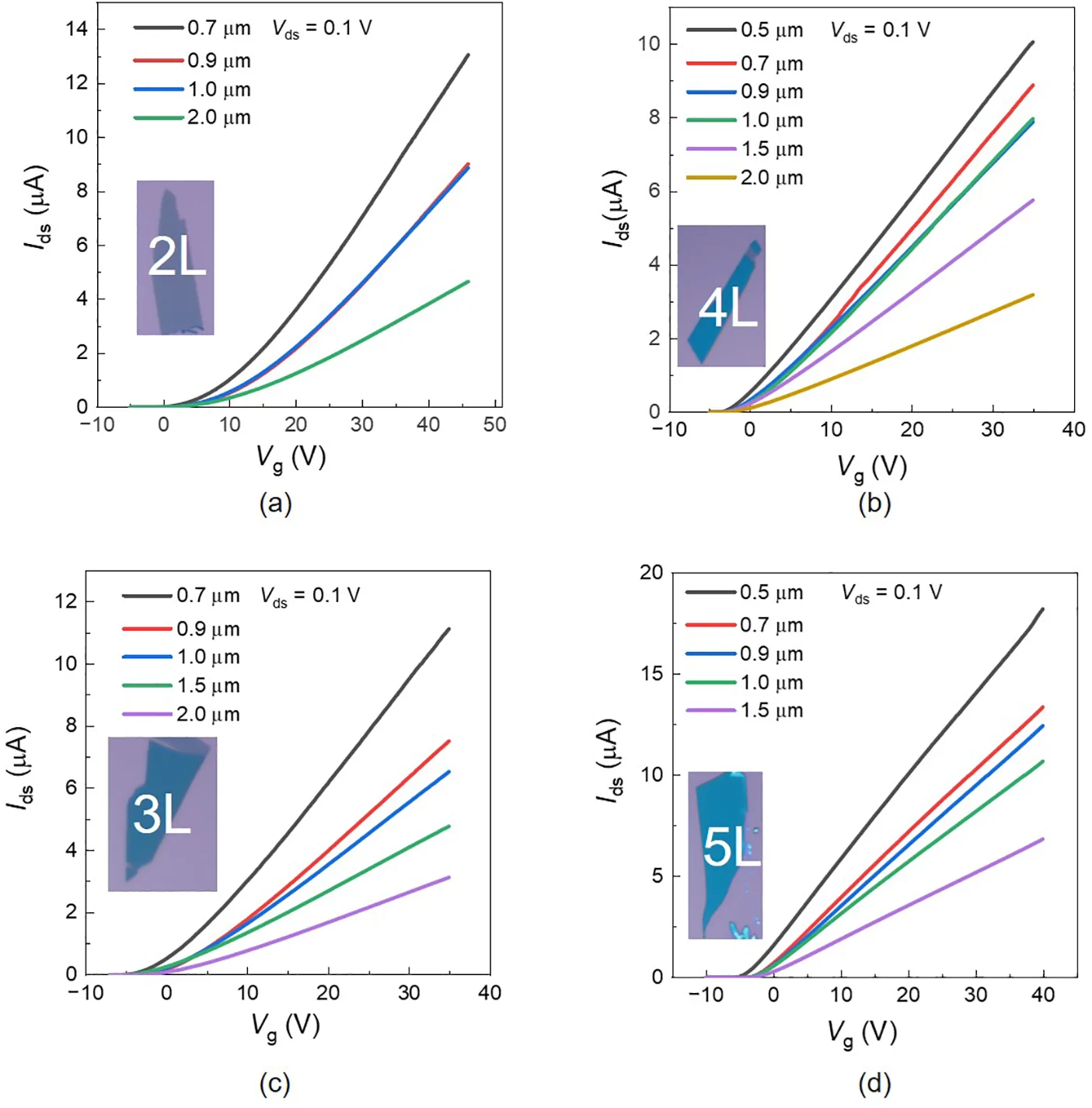
Multilayer TLM devices with 90 nm SiO_2_ as the gate dielectric. (a), (b), (c), (d) Transfer characteristics of 2‑ to 5‑layer MoS_2_ devices; insets show optical images of the multilayer MoS_2_ before device fabrication.

**Fig 7:**
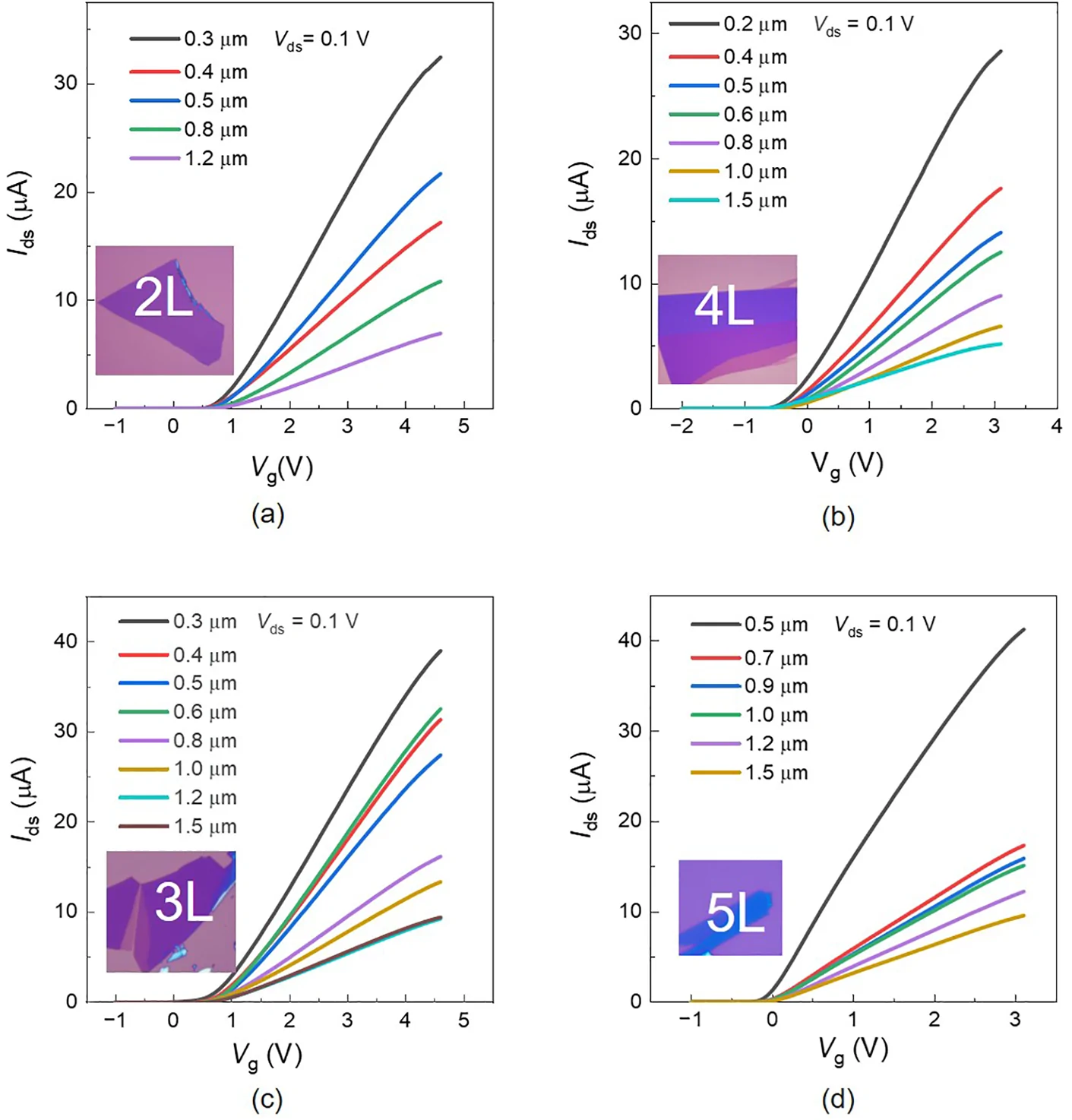
Multilayer TLM devices with 10 nm HfO_2_ as the gate dielectric. (a), (b), (c), (d) Transfer characteristics of 2‑ to 5‑layer MoS_2_ devices; insets show optical images of the multilayer MoS_2_ before device fabrication.

**Fig 8:**
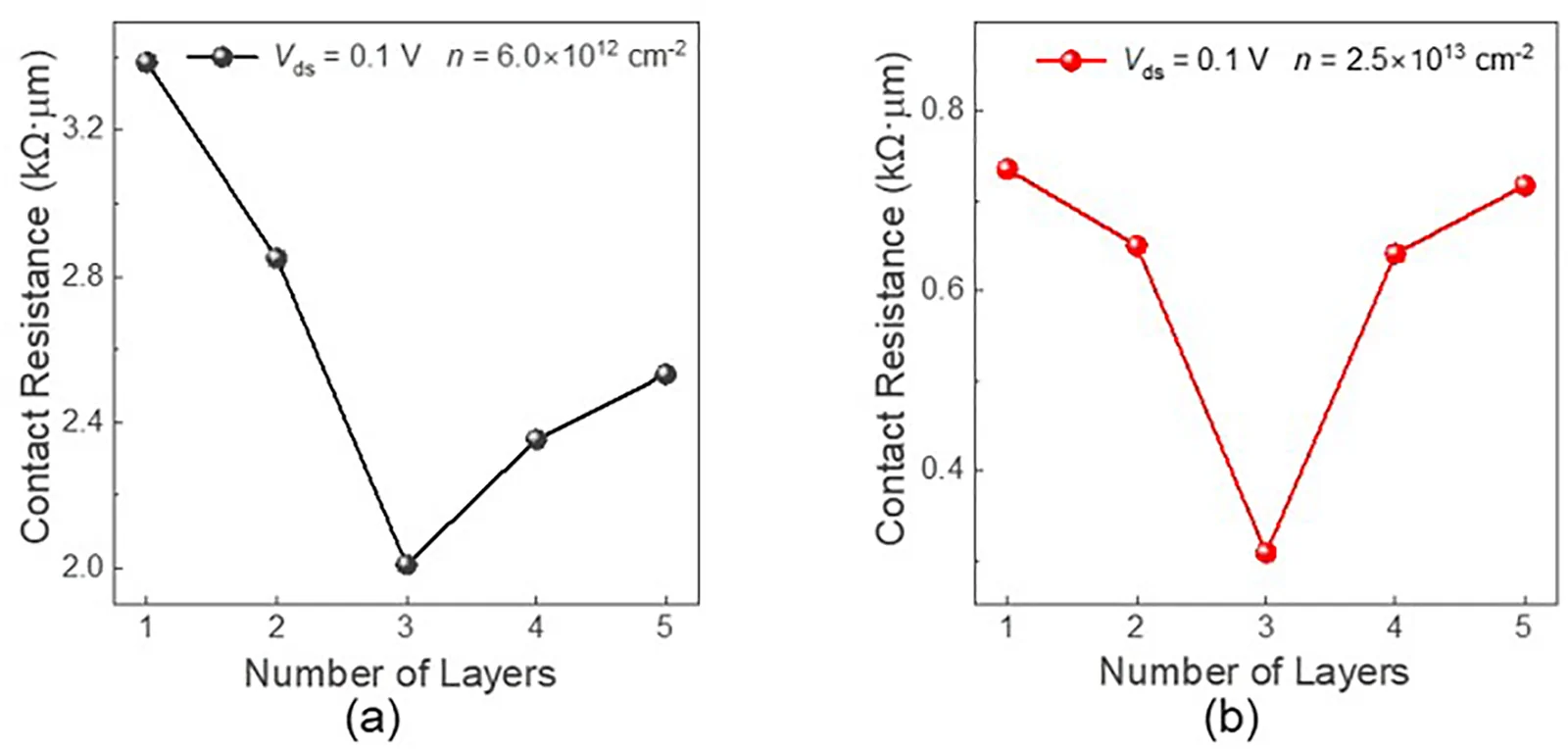
Influence of the 90 nm SiO_2_ and 10 nm HfO_2_ on contact resistance. (a) Variation of contact resistance with MoS_2_ layer number for devices with SiO_2_ as the gate dielectric, measured at a carrier concentration of 6 × 10^12^ cm^−2^. The inset illustrates a schematic of the device structure. (b) Variation of contact resistance with MoS_2_ layer number for devices with HfO_2_ as the gate dielectric, measured at a carrier concentration of 2.5 × 10^13^ cm^−2^. The inset illustrates a schematic of the corresponding device structure.

Notably, at higher carrier concentrations, the increase in the contact resistance of HfO_2_-based devices within the range of 3–5 layers is significantly greater than that of SiO_2_ back-gated devices. To exclude the influence of carrier concentration differences, we compared the two types of devices at the same carrier concentration (Fig 9 (a)). The results show that when the concentrations are the same, the trends of the layer number changes of the two are highly consistent. On the one hand, this confirms the universality of the rule that “the contact resistance of MoS_2_-Sb first decreases and then increases with the number of layers”. These results indicate that the gate dielectric primarily influences the absolute magnitude of the contact resistance through electrostatic modulation, whereas the layer-dependent evolution of the contact resistance is governed predominantly by the intrinsic electronic structure and interlayer transport properties of MoS_2_.

**Fig 9:**
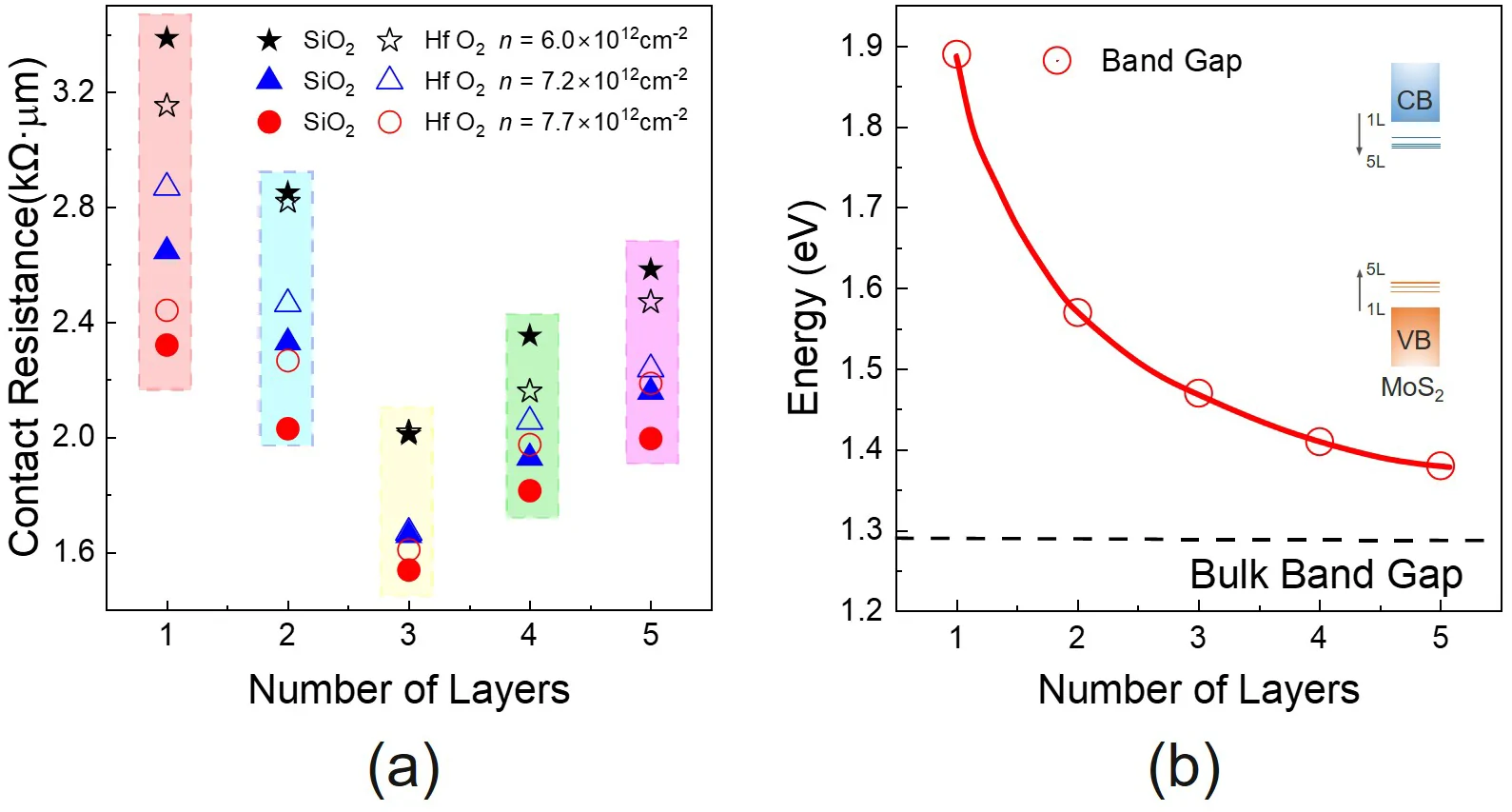
Influence of carrier concentration differences on MoS_2_ layers. (a) Comparison of contact resistance for devices fabricated with two different gate dielectrics under the same carrier concentration (b) Layers-dependent variation of the bandgap in MoS_2_.

For the MoS_2_ material, from the perspective of the energy band structure, monolayer MoS_2_ exhibits as a direct – bandgap semiconductor (approximately 1.9 eV) due to the breaking of spatial inversion symmetry as shown in Fig 9 (b). Both the bottom of the conduction band and the top of the valence band are located at the K point. As the number of layers increases, the inter – layer coupling effect strengthens. The top of the valence band gradually shifts from the K point to the Γ point, and the system transforms into an indirect-bandgap semiconductor [29]. Moreover, the bandgap decreases with the increase in the number of layers (from 1.90 eV to 1.51 eV within 1–3 layers) [30] as shown in Fig 9 (b).

The gate dielectric influences the electrical characteristics of the MoS_2_ field-effect transistor primarily through its electrostatic coupling to the channel. Compared with the conventional 90 nm SiO_2_ dielectric, the 10 nm HfO_2_ dielectric possesses a significantly higher dielectric constant, resulting in stronger gate electrostatic control and more efficient carrier accumulation in the MoS_2_ channel. Consequently, the depletion width at the Sb–MoS_2_ interface is reduced, facilitating electron injection and lowering the measured contact resistance.

In addition to enhanced electrostatic coupling, the high-κ dielectric also modifies the local electrostatic environment through dielectric screening, which weakens the Coulomb interaction between charged impurities and channel carriers. This effect contributes to improved carrier transport under high carrier concentrations. On the other hand, high-κ dielectrics may introduce remote optical phonon scattering and interface trap states, both of which can degrade carrier mobility. Therefore, the experimentally observed contact resistance represents the combined influence of carrier injection at the metal–semiconductor interface and carrier transport within the channel.

To distinguish dielectric effects from intrinsic thickness-dependent behavior, the contact resistance obtained from SiO_2_- and HfO_2_-based devices was further compared at identical carrier concentrations. As shown in Fig 9(a), both device platforms exhibit nearly identical layer-dependent trends, with the minimum contact resistance consistently occurring for trilayer MoS_2_. This observation indicates that although the gate dielectric modifies the absolute magnitude of the contact resistance by altering the electrostatic environment, it does not change the intrinsic physical mechanism governing the non-monotonic dependence of contact resistance on MoS_2_ thickness. Instead, the observed behavior is primarily determined by the competition between Schottky barrier modulation associated with thickness-dependent band structure evolution and the increasing interlayer transport resistance in multilayer MoS_2_.

In the range of 1–3 layers, the significant narrowing of the bandgap reduces the Schottky barrier, which dominates the downward trend of the contact resistance. Starting from the third layer, the change in the bandgap becomes gentle, and the barrier regulation effect gradually weakens. Meanwhile, as the number of layers increases, carriers need to cross more van der Waals interfaces during the transport process, and the introduced intrinsic inter-layer resistance (*R*_Int_) begins to accumulate [31,32]. This resistance stems from the weak van der Waals coupling between layers and the limited probability of inter-layer carrier transition [33]. Its magnitude is mainly determined by the electronic structure of the material itself and the inter-layer interaction, and it basically does not change with the carrier concentration.

This competitive mechanism between “energy band regulation” and “inter-layer transport” well explains the non – monotonic layer – dependent behavior observed in experiments and reaches a dynamic balance around 3 layers, resulting in the contact resistance reaching a minimum value at this number of layers. In addition, this model can also reasonably explain the difference in the rising amplitude of the contact resistance in the range of 3–5 layers under different gate dielectrics (SiO_2_ and HfO_2_): at a higher carrier concentration, the component of the contact resistance dominated by the barrier becomes lower, while the intrinsic inter – layer resistance (*R*_Int_) is basically not regulated by the external carrier concentration. Therefore, in devices with a high carrier concentration, the reduced variation amplitude of the barrier – related resistance makes the relative contribution of the inter – layer resistance in the total resistance more prominent, leading to a more significant upward trend of the contact resistance with the increase in the number of layers in MoS_2_ above 3 layers.

It is crucial to note that in the range of 1–3 layers, the reduction in contact resistance cannot be explained solely by bandgap narrowing. From an electronic structure perspective, the thickness-dependent bandgap evolution of MoS_2_ is accompanied by a shift in both the valence band maximum and conduction band minimum. In particular, previous studies have reported a downward shift of the conduction band minimum with increasing layer number, resulting in an increase in electron affinity.

This increase in electron affinity effectively lowers the Schottky barrier height for electron injection at the MoS_2_–Sb interface, thereby facilitating carrier transport and reducing contact resistance. In addition, the weakening of quantum confinement in few-layer MoS_2_ further enhances carrier injection efficiency. Therefore, the observed decrease in contact resistance from monolayer to trilayer MoS_2_ originates from the combined effects of conduction band alignment and reduced quantum confinement, rather than bandgap reduction alone.

### 3.1 Quantitative interpretation of thickness-dependent contact resistance

Although direct extraction of the Schottky barrier height was beyond the scope of the present study, the experimentally observed non-monotonic variation of contact resistance can be interpreted quantitatively by considering the thickness-dependent evolution of the MoS_2_ electronic structure. As summarized in Table 3, the bandgap decreases significantly from 1.90 eV for monolayer MoS_2_ to 1.47 eV for trilayer MoS_2_, followed by only a slight reduction for thicker layers. The reduction in bandgap is accompanied by a downward shift of the conduction band minimum and an increase in electron affinity, which lowers the electron injection barrier at the Sb–MoS_2_ interface and facilitates carrier transport. Moreover, the calculated Schottky barrier heights *Φ*_B_ decrease systematically from 19.44 meV for monolayer MoS_2_ to 5.41 meV for five-layer MoS_2_, supporting the proposed reduction in carrier injection barrier with increasing thickness. They are further illustrated in Fig 10.

**Table 3:** Thickness-dependent evolution of the electronic structure of MoS_2_ used for the analytical interpretation.

MoS_2_ Layer Number	Bandgap (eV)	Estimated *Φ*B (meV)	Dominant Effect on Contact Transport
1L	1.90	19.44	Large bandgap, relatively high Schottky barrier
2L	1.57	15.54	Reduced bandgap lowers carrier injection barrier
3L	1.47	12.62	Optimum balance between barrier reduction and interlayer transport
4L	1.41	8.93	Further bandgap reduction becomes small while interlayer transport resistance increases
5L	1.38	5.41	Interlayer transport dominates carrier injection

**Fig 10:**
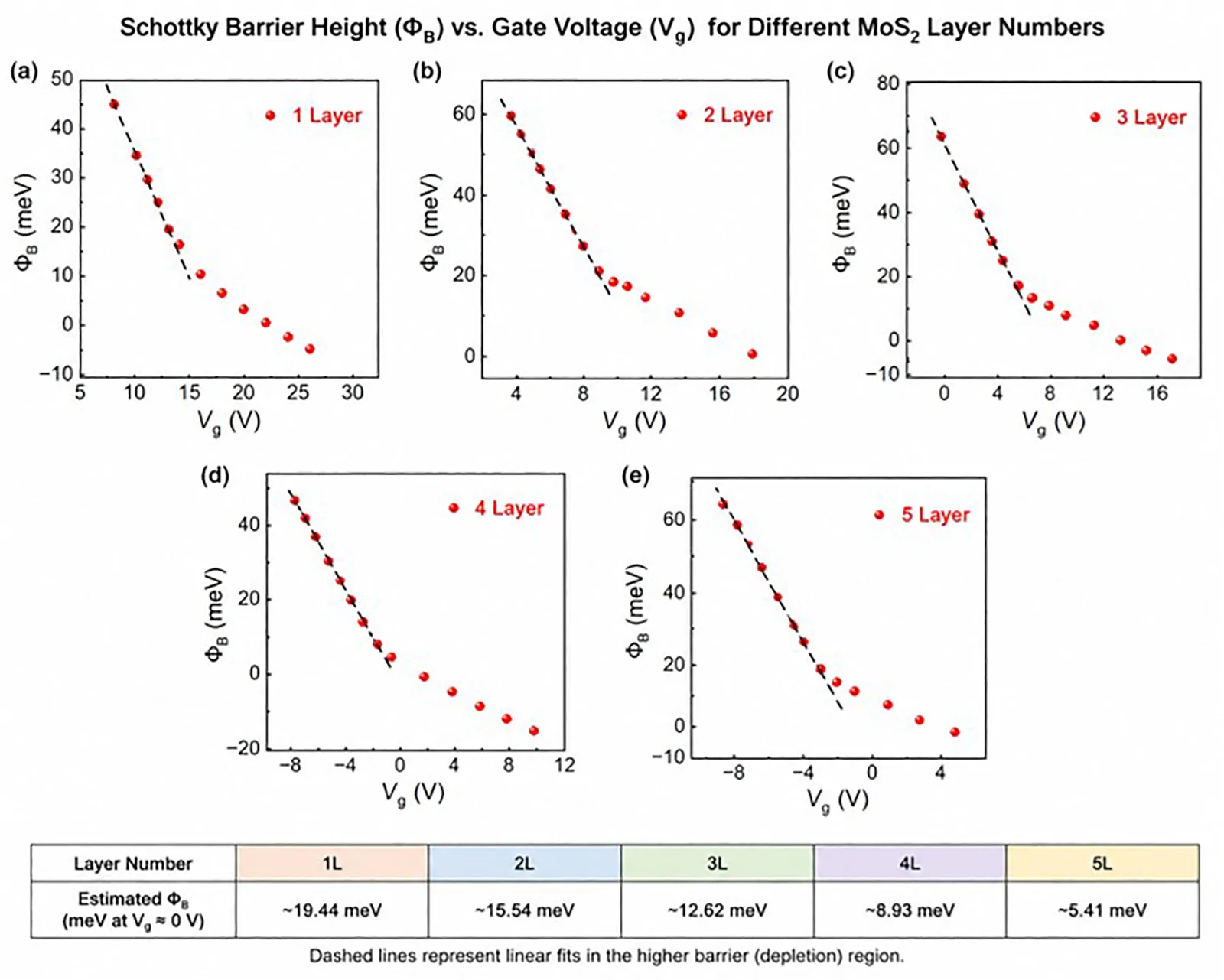
Schottky barrier height (Φ_B_) as a function of gate voltage (V_g_) for Sb-contacted MoS_2_ devices with different layer number.

To further quantify the physical origin of the observed non-monotonic contact resistance, the total contact resistance can be expressed as the combined contribution of the Schottky barrier resistance and the intrinsic interlayer transport resistance (as illustrated in Fig 11):

**Fig 11:**
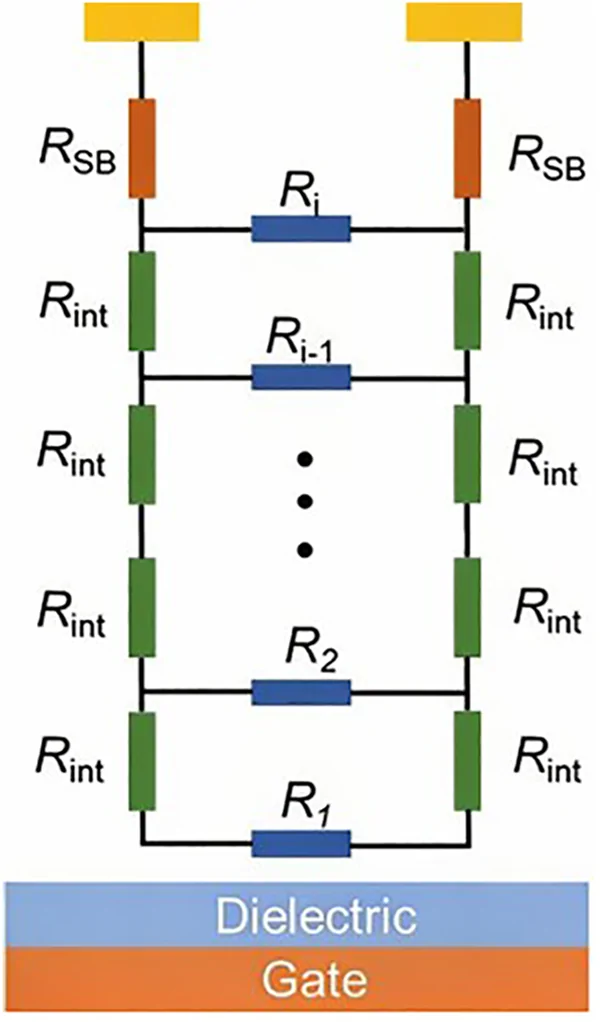
Interlayer resistance model.


$$\begin{array}{c} R_{C}=\;R_{SB}+R_{Int} \end{array}$$ (4)

where $R_{SB}$ represents the carrier injection resistance associated with the Schottky barrier at the MoS_2_-Sb interface and $R_{Int}$ denotes the intrinsic resistance arising from carrier transport across adjacent MoS_2_ layers.

In multilayer MoS_2_ devices, carrier transport beneath the metal contact is inherently 3D. Electrons injected from the Sb electrode initially enter the uppermost MoS_2_ layer directly beneath the contact and subsequently propagate through adjacent layers before reaching the conducting channel. Because neighboring MoS_2_ layers are coupled only through weak van der Waals interactions, the out-of-plane conductivity is substantially lower than the in-plane conductivity. Consequently, an additional interlayer transport resistance is introduced, which increases with the number of layers participating in the vertical transport path.

Furthermore, carrier injection is concentrated near the contact edge, leading to current crowding in the vicinity of the metal–semiconductor interface. This effect reduces the effective current injection area and becomes increasingly important as the vertical transport path becomes longer. Therefore, although increasing MoS_2_ thickness decreases the Schottky barrier height through bandgap narrowing, the simultaneous increase in interlayer transport resistance and current crowding limits further improvement in carrier injection. These competing mechanisms explain the experimentally observed non-monotonic dependence of contact resistance on MoS_2_ thickness.

As the MoS_2_ thickness increases from monolayer to trilayer, the bandgap decreases from 1.90 eV to 1.47 eV, accompanied by a downward shift of the conduction band minimum and an increase in electron affinity. Consequently, the Schottky barrier height is reduced, resulting in enhanced carrier injection and a decrease in $R_{SB}$. However, further increasing the number of layers introduces additional van der Waals interfaces, leading to an increase in the intrinsic interlayer transport resistance $R_{Int}$. Therefore, while $R_{SB}$ continues to decrease only gradually beyond three layers, the increasing contribution of $R_{Int}$ becomes dominant, giving rise to the experimentally observed increase in the overall contact resistance.

This analytical model is consistent with the experimentally measured contact resistance and provides a quantitative physical interpretation of the transition from barrier-dominated transport in thin MoS_2_ to interlayer-resistance-dominated transport in thicker multilayer structures. Although the proposed transport mechanism is supported by the analytical Schottky barrier estimation and the observed thickness-dependent electrical characteristics, direct extraction of the Schottky barrier height through temperature-dependent current–voltage measurements was not performed in the present work. Temperature-dependent transport measurements provide a powerful method for distinguishing thermionic emission from tunneling transport and for quantitatively evaluating Schottky barrier modulation. Such measurements would provide additional experimental verification of the proposed carrier injection mechanism and will be pursued in future investigations.

In addition to Schottky barrier modulation, the electrical characteristics of metal–MoS_2_ contacts are strongly influenced by Fermi-level pinning arising from interface states and metal-induced gap states (MIGS). These interface states reduce the sensitivity of the Schottky barrier height to the metal work function, limiting the effectiveness of conventional contact engineering. Owing to its semimetallic electronic structure and relatively low density of states near the Fermi level, Sb is expected to form a weakly interacting van der Waals interface with MoS_2_, thereby suppressing MIGS and partially mitigating Fermi-level pinning. As a result, carrier injection is improved and the contact resistance is reduced. Although the present results are consistent with this physical picture, quantitative determination of the Fermi-level pinning factor requires systematic investigation using multiple contact metals with different work functions and is therefore beyond the scope of the present study.

The non-monotonic thickness dependence observed in the present work is consistent with previously reported transport models for metal–MoS_2_ contacts, in which increasing MoS_2_ thickness simultaneously reduces the Schottky barrier through bandgap narrowing while increasing the resistance associated with vertical interlayer carrier transport. The analytical interpretation presented here therefore follows the same underlying physical principles reported for two-dimensional semiconductor contacts. Nevertheless, the optimum layer thickness is expected to depend on the specific contact material, dielectric environment, carrier concentration, and fabrication conditions. Consequently, the present results should be regarded as a physically consistent interpretation for Sb-contacted MoS_2_ devices rather than a universal scaling law applicable to all contact systems. The contact resistance values summarized in Table 1, together with the analytical discussion presented above, indicate that the observed thickness dependence follows the same physical trends reported for representative low-resistance metal and semimetal contacts to MoS_2_.

### 3.2 Robustness of the thickness-dependent contact resistance under different carrier concentrations

To further evaluate the robustness of the observed thickness-dependent contact resistance, additional electrical measurements were performed at different electrostatically induced carrier concentrations for both the 90 nm SiO_2_ and 10 nm HfO_2_ devices. The extracted contact resistance values are summarized in Tables 4 and 5. Increasing the carrier concentration systematically reduces the absolute contact resistance because of enhanced carrier accumulation and more efficient carrier injection at the MoS_2_-Sb interface. Importantly, despite the variation in carrier concentration, the minimum contact resistance is consistently observed for trilayer MoS_2_ for both dielectric platforms. This consistency indicates that the non-monotonic dependence of contact resistance on layer number is robust over a broad range of carrier concentrations and is not limited to a single electrostatic operating condition.

**Table 4:** Contact resistance of Sb-contacted MoS_2_ devices on 90 nm SiO_2_ at different carrier concentrations.

Layer	$R_{𝐜}\;(\Omega )$ at n = 6 × 10^12^ cm^−2^	$R_{𝐜}\;(\Omega )$ at n = 7.2 × 10^12^ cm^−2^	$R_{𝐜}\;(\Omega )$ at n = 7.7 × 10^12^ cm^−2^	Mean ± SD (Ω·μm)
**1L**	3387	2647	2321	2785.0 ± 546.2
**2L**	2850	2330	2030	2403.3 ± 414.9
**3L**	2010	1660	1539	1736.3 ± 244.6
**4L**	2353	1929	1815	2032.3 ± 283.5
**5L**	2532	2158	1996	2228.7 ± 274.9

**Table 5:** Contact resistance of Sb-contacted MoS_2_ devices on 10 nm HfO_2_ at different carrier concentrations.

Layer	${𝐑}_{𝐜}\;(\Omega )$ at n = 6 × 10^12^ cm^−2^	${𝐑}_{𝐜}\;(\Omega )$ at n = 7.2 × 10^12^ cm^−2^	${𝐑}_{𝐜}\;(\Omega )$ at n = 7.7 × 10^12^ cm^−2^	${𝐑}_{𝐜}\;(\Omega )$ at n = 2.5 × 10^13^ cm^−2^	Mean ± SD (Ω·μm)
**1L**	3152	2868	2442	735	2299.2 ± 1082.9
**2L**	2819	2464	2267	650	2050.0 ± 960.9
**3L**	2018	1669	1610	308	1401.2 ± 750.7
**4L**	2160	2055	1975	641	1707.8 ± 715.2
**5L**	2470	2237	2188	717	1903.0 ± 800.2

For completeness, the mean values and standard deviations of the measured contact resistance are also presented in Tables 4 and 5. These statistical quantities summarize the variation associated with different carrier concentrations measured on the same representative device for each layer number. They are provided to illustrate the consistency of the thickness-dependent trend under different electrostatic conditions and should not be interpreted as statistical reproducibility across independently fabricated devices. Comprehensive statistical validation using multiple fabricated devices remains an important direction for future investigation.

In practical device applications, the environmental stability of the contact metal is an important consideration. In the present devices, the Sb contact layer was immediately covered by a 40 nm Ag capping layer following deposition. In addition to reducing the series resistance of the contact, the Ag overlayer minimizes direct exposure of Sb to the ambient environment and therefore helps suppress surface oxidation during device fabrication and electrical characterization. Nevertheless, prolonged environmental exposure may still influence the electrical characteristics through gradual oxidation or interface modification. Comprehensive long-term stability and reliability studies, including ambient aging and thermal stress tests, are beyond the scope of the present work but represent an important direction for future investigation.

## 4 Conclusion

This work systematically investigated the thickness dependence of the contact resistance in Sb-contacted MoS_2_ field-effect transistors fabricated on both 90 nm SiO_2_ and 10 nm HfO_2_ gate dielectrics. A non-monotonic dependence of contact resistance on MoS_2_ thickness was consistently observed, with trilayer MoS_2_ exhibiting the lowest contact resistance under the investigated experimental conditions. The observed behavior was interpreted through the competing influences of Schottky barrier reduction associated with thickness-dependent bandgap narrowing and the increasing interlayer transport resistance in multilayer MoS_2_.

Additional analyses, including analytical Schottky barrier estimation, comprehensive TLM fitting validation, comparison over different carrier concentrations, and expanded discussion of dielectric effects and interface transport, further support the proposed physical interpretation. These results indicate that semimetallic Sb provides a promising contact material for low-resistance MoS_2_ devices while also highlighting the importance of channel thickness optimization.

It should be noted that the optimum layer number and absolute contact resistance may depend on fabrication conditions, interface quality, dielectric environment, carrier concentration, and contact material. Therefore, the present findings should be interpreted as applying to the investigated MoS_2_-Sb contact system rather than as a universal scaling behavior. Future work will include multiple-device statistical validation, temperature-dependent transport measurements, detailed interface characterization, and long-term reliability studies to further establish the general applicability of the proposed transport mechanism.
